# Olodaterol Attenuates Citric Acid-Induced Cough in Naïve and Ovalbumin-Sensitized and Challenged Guinea Pigs

**DOI:** 10.1371/journal.pone.0119953

**Published:** 2015-03-17

**Authors:** Eva Wex, Thierry Bouyssou

**Affiliations:** Boehringer Ingelheim Pharma GmbH & Co. KG, Respiratory Diseases Research, Biberach a. d. Riss, Germany; Central South University, CHINA

## Abstract

Excessive coughing is a common feature of airway diseases. Different G-protein coupled receptors, including β2-adrenergic receptors (β2-AR), have been implicated in the molecular mechanisms underlying the cough reflex. However, the potential antitussive property of β2-AR agonists in patients with respiratory disease is a matter of ongoing debate. The aim of our study was to test the efficacy of the long-acting β2-AR agonist olodaterol with regard to its antitussive property in a pre-clinical model of citric acid-induced cough in guinea pigs and to compare the results to different clinically relevant β2-AR agonists. In our study β2-AR agonists were intratracheally administered, as dry powder, into the lungs of naïve or ovalbumin-sensitized guinea pigs 15 minutes prior to induction of cough by exposure to citric acid. Cough events were counted over 15 minutes during the citric acid exposure. Olodaterol dose-dependently inhibited the number of cough events in naïve and even more potently and with a greater maximal efficacy in ovalbumin-sensitized guinea pigs (p < 0.01). Formoterol and salmeterol showed a trend towards reducing cough. On the contrary, indacaterol demonstrated pro-tussive properties as it significantly increased the number of coughs, both in naïve and ovalbumin-sensitized animals (p < 0.001). In conclusion, olodaterol, at doses eliciting bronchodilation, showed antitussive properties in a model of citric acid-induced cough in naïve and ovalbumin-sensitized guinea pigs. This is in agreement with pre-clinical and clinical studies showing antitussive efficacy of β2-AR agonists. Indacaterol increased the number of coughs in this model, which concurs with clinical data where a transient cough has been observed after indacaterol inhalation. While the antitussive properties of β2-AR agonists can be explained by their ability to lead to the cAMP-induced hyperpolarization of the neuron membrane thereby inhibiting sensory nerve activation and the cough reflex, the mechanism underlying the pro-tussive property of indacaterol is not known.

## Introduction

While under physiological conditions cough is an essential reflex mechanism that helps to clear the lungs from particles and secretions and thus, protects the lung against the inhalation of harmful irritants, it can become chronic and debilitating under pathological conditions [1]. Excessive coughing is a common feature of airway diseases, like chronic obstructive pulmonary disease (COPD) [2]. In COPD daily cough is reported to be a predictive factor for frequent exacerbations [3,4]. COPD patients, show an increased responsiveness to capsaicin indicating the existence of an increased cough reflex sensitivity rather than cough being simply a consequence of the increased airway secretion [5,6]. Pharmacological interventions are currently confined to treating the cause of cough, but treatment options controlling the cough response per se with an acceptable therapeutic ratio are presently not available [7]. Since chronic cough is not only an disturbing symptom of respiratory diseases which affects the health-related quality of life but might also induce the release of pro-inflammatory mediators due to repetitive mechanical and physical stress on the airway cells, reducing cough might also modulate disease progression [8].

A variety of G-protein coupled receptors (GPCRs), including β_2_-adrenergic receptors (β_2_-ARs), have been implicated in the molecular mechanisms underlying the cough reflex [9]. Freund-Michel et al. have shown that β_2_-AR agonists possess antitussive properties in guinea pigs through direct inhibition of sensory nerve activity, independent of bronchodilation [10]. According to their results activation of β_2_-ARs inhibits the depolarization of the vagus nerve by stimulating adenylyl cyclase to synthesize cyclic-3’,5’-adenosine monophosphate (cAMP). The accumulation of cAMP causes the protein kinase G (PKG)-mediated opening of large conductance calcium-activated potassium channels (BK_Ca_) which induces hyperpolarization of the neuron membrane thereby inhibiting sensory nerve activation and the cough reflex. Besides the fact that β_2_-AR agonists have been used for years as bronchodilators in the treatment of airway diseases still controversy on their antitussive properties remains. While some clinical studies found beneficial effects of β_2_-AR agonists on cough [11–17] others failed to show this correlation [18–22].

Olodaterol (Striverdi) is a novel once-daily, long-acting β_2_-adrenergic receptor agonist. Olodaterol recently received approval for the once-daily maintenance treatment of COPD in the USA, Canada, Russia and many European countries [23]. Olodaterol shows a potent, nearly full agonistic response at the hβ_2_-AR (EC_50_ = 0.1nM; intrinsic activity = 88% compared to isoproterenol) and a significant selectivity profile (241-fold and 2299-fold towards the hβ_1_- and hβ_3_-ARs, respectively) [24].

The aim of our study was to test the efficacy of olodaterol with regard to its antitussive property in a model of citric acid-induced cough in guinea pigs and to compare the results to different clinically relevant β_2_-AR agonists. The compounds were tested after intratracheal dry powder application in naïve animals, as well as in ovalbumin (OVA)-sensitized animals to simulate the increased cough reflex sensitivity found in asthmatic patients.

## Materials and Methods

### 1.1. Reagents and test compounds

Ovalbumin (Serva Electrophoresis GmbH, Heidelberg, Germany) was dissolved and diluted in phosphate buffered saline (PBS; Lonza, Verviers, Belgium) for the immunization, and in 0.9% saline (Delta Select GmbH, Dreieich, Germany) for the OVA challenge. Aluminium hydroxide (ImjectAlum; ThermoScientific, Rockford, IL, USA) was dissolved and diluted in PBS. Citric acid monohydrate and pyrilamine maleate were purchased from Sigma-Aldrich Chemie GmbH (Steinheim, Germany) and dissolved and diluted in 0.9% saline. Respitose (Lactose monohydrate, ML003) was from Boehringer Ingelheim (Ingelheim, Germany). Codeine phosphate hemihydrate was purchased from Merck Company (Germany). Olodaterol hydrochloride, salmeterol xinafoate, formoterol fumarate, and indacaterol maleate were synthesized at the Department of Chemical Research, Boehringer Ingelheim Pharma GmbH & Co. KG (Biberach, Germany).

### 1.2. Animals

Male albino Dunkin-Hartley guinea pigs (350–550 g) were purchased from Charles River (Charles River WIGA GmbH, Sulzfeld, Germany). Animals were kept in rooms maintained at constant temperature (22°C ± 2°C) and humidity (60% ± 15%) under a 12 h light-dark cycle. The animals were housed in groups of 5–6 in solid floor cages and allowed free access to water and standard food.

### 1.3. Ethics statement

All animal experimentation was conducted in strict accordance with German national guidelines and legal regulations. The protocol was approved by the ethical committee of the Regierungspräsidium Tübingen (Germany) (Permit Number: 08–004, 10–008 and 12–020). All surgery was performed under sodium pentobarbital anesthesia, and all efforts were made to minimize suffering.

### 1.4. Compound application

For administration of β_2_-AR agonists or codeine in the bronchoconstriction or cough experiments a powder inhaler device (DP-4 Insufflator, Penn-Century Inc., Wyndmoor, PA, USA) was used. The test compound was formulated with Respitose and 10 mg of Respitose containing the respective amount of test compound was delivered intratracheally to each animal under short-term isoflurane (4.5% v/v) (Forene, Abbott GmbH and Co. KG, Wiesbaden, Germany) anaesthesia 15 minutes prior to citric acid exposure. The β_2_-AR antagonist, ICI-118,551, was administered intraperitoneally at 0.5 mg/kg 30 minutes before intratracheal indacaterol application.

### 1.5. Model of acetylcholine-induced bronchoconstriction in anaesthetized guinea pigs

The bronchoprotection by β_2_-AR agonist pre-treatment was measured as described previously [24]. Anaesthesia of guinea pigs was induced by i.p. injection of 50 mg/kg pentobarbital followed by intravenous (i.v.) infusion of pentobarbital (15 mg/kg/h) via the jugular vein. A tracheal cannula was introduced for artificial ventilation, and the internal jugular vein was cannulated for acetylcholine injection. Animals were ventilated at a stroke volume of 10 ml/kg and a rate of 60 strokes per minute. Bronchoconstriction was recorded by using a modified version of the method of Konzett-Roessler [25] as an increase in airflow pressure (cm of H_2_O). After three stable acetylcholine-induced bronchospasms, compounds were administered. To address the onset of action of the compounds, the β_2_-AR agonists were dissolved in a mixture of distilled water and ethanol (40:60, v/v) at concentrations permitting the administration of the desired dose with one actuation of the Respimat Soft Mist inhaler (Boehringer Ingelheim, Ingelheim, Germany) connected to an endotracheal tube. Bronchoconstrictions were induced by acetylcholine (10 μg/kg i.v.) 1, 3, 5, 7, 10 and 20 minutes after drug inhalation. To address the efficacy, the test compound was given as a dry powder with Respitose as described in paragraph 1.4. Guinea pigs regained consciousness after drug administration and they were anaesthetized two hours later in order to perform acetylcholine-induced bronchospasm as described above. The bronchodilatory activity of the different β_2_-AR agonists was tested against increasing doses of acetylcholine (2–20 μg/kg i.v., increment of 2 μg/kg between two doses) injected every 10 minutes for 90 minutes.

### 1.6. Model of citric acid-induced cough in conscious guinea pigs

Guinea pigs were starved overnight (12 h) but had free access to drinking water. Conscious guinea pigs were individually exposed to an aerosol of citric acid (citric acid monohydrate dissolved in 0.9% saline) in whole body inhalation boxes. The nebulizer head was connected to the Aeroneb nebulizer system (Aerogen, Ireland) and filled with 10 ml citric acid (0.4 M). Citric acid was nebulized at a rate of 0.19 ml/min for 15 minutes (particle size 2.5 to 4 μm). Cough events were counted over 15 minutes during the citric acid exposure.

### 1.7. Measurement of cough responses

Cough was recorded through the Buxco plethysmograph system suitable for conscious rats and guinea pigs (Buxco Research Systems, Wilmington, NC, USA). Conscious animals were placed individually into a transparent plastic whole body plethysmograph chamber (5 l). The baseline air flow was recorded for 2 minutes before initiating the citric acid challenge. Animals’ welfare was continuously monitored and the number of coughs was counted for 15 minutes during citric acid exposure. Cough was detected by the Buxco software as a transient increase in airflow (a rapid inspiration followed by rapid expiration), over the normal flow (ml/s) caused by a quick large abdominal movement. A bias flow generator supplied air to each chamber at a rate of 2 l min^-1^ and withdrew air at a rate of 2.5 l min^-1^. The Buxco Cough Analyser utilized a specific algorithm to count cough in 15 minutes by recognition of a box flow waveform that crosses a positive threshold to a negative one within a maximum time period. Using these criteria together, cough was easily distinguished from sneezes, augmented breaths and movements. Additionally, cough recording was validated by visual confirmation of cough events. At the end of the experiments, animals were euthanized by an overdose of pentobarbital (2 ml/kg) (Narcoren, Merial GmbH, Hallbergmoos, Germany).

### 1.8. OVA sensitization and measurement of OVA-specific IgE in serum

Animals were immunized on day 1 and 2 by subcutaneous injection of 0.5 ml per animal of a solution of OVA (40 μg/ml) and aluminium hydroxide [Al(OH)_3_]. On day 15 animals were exposed to an OVA aerosol (1.25 mg/ml) for 5 minutes using a Dräger Inhalette (Dräger Medical AG & Co. KGaA, Lübeck, Germany). Pyrilamine maleate (2 mg/kg) was injected intraperitoneally (i.p.) 30 minutes before exposure to OVA aerosol to avoid any lethal bronchospasm due to histamine release from mast cell degranulation. Cough experiments were performed 24 hours after OVA aerosol inhalation.

For measurement of OVA-specific IgE guinea pigs were euthanized with an intraperitoneal injection of pentobarbital (2 ml/kg) (Narcoren, Merial GmbH, Hallbergmoos, Germany) 24 h after OVA aerosol inhalation. Immediately after, blood was collected. After coagulation the serum was separated by centrifugation for 5 min at 10,000 rpm, 4°C (Microcentrifuge 5415R, Eppendorf, Germany). The serum was then aspirated off and stored at -20°C until analysed. A guinea pig ovalbumin-specific IgE (OVA sIgE) ELISA kit (Cusabio Biotech Co. Ltd, China) was used to analyse IgE levels in serum. The protocol for this assay was outlined by the manufacturer.

### 1.9. Statistical analyses

For the comparison vehicle-treated, naïve animals vs. compound-treated, naïve animals a one-way ANOVA with Fisher’s LSD post-hoc test was applied. A method for ordered alternatives was used to adjust for multiple testing. Assuming an increasing effect with increasing dose the highest dose was tested first and if and only if a statistically significant difference could be shown, the comparison to the lower doses followed. OVA-treated animals were compared with the one-way ANOVA followed by the Dunnett’s multiple comparisons test. Data were expressed as a mean ± S.E.M. The limit of the significance was taken as *p* values less than 0.05 (*p* < 0.05). The half-maximal effective dose (ED_50_) values were calculated with 95% confidence intervals by a nonlinear regression analysis. The percentage inhibition or increase was calculated from the mean number of coughs according to: % inhibition or increase = 100-(Y/K1)*100. With K1 being the mean number of coughs of vehicle-treated control animals and Y being the mean number of coughs of compound-treated animals. These tests were performed using GraphPad Prism version 6.01 for Windows, GraphPad Software, La Jolla, California, USA, “www.graphpad.com”.

## Results

### 2.1. Time to onset of action and therapeutic dose of β_2_-adrenergic agonists in a model of acetylcholine-induced bronchoconstriction in anaesthetized guinea pigs

**Fig 1:**
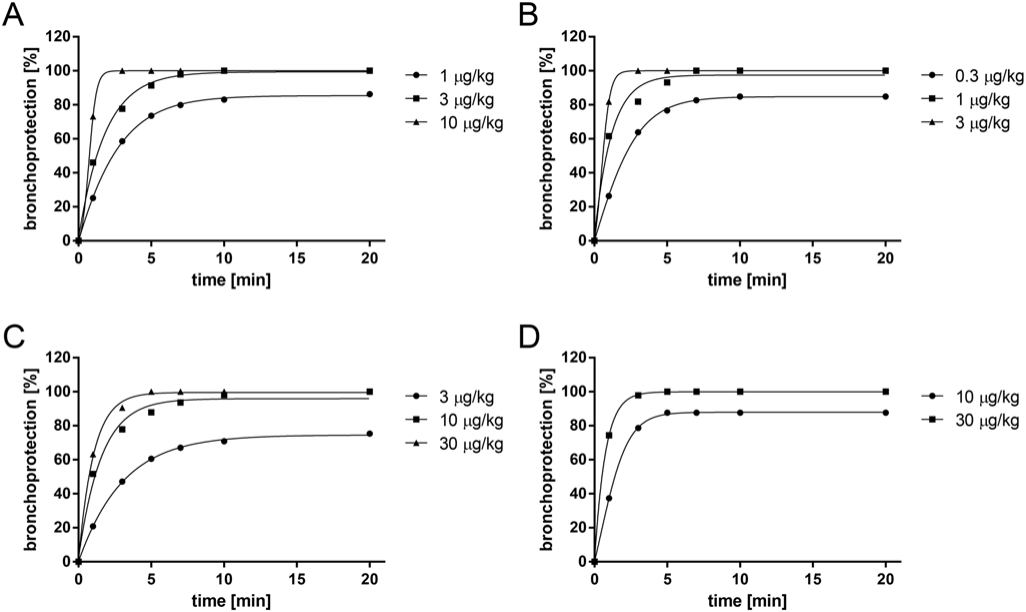
Time to onset of action of β_2_-agonists in the acetylcholine-induced bronchoconstriction model in guinea pigs.

**Fig 2:**
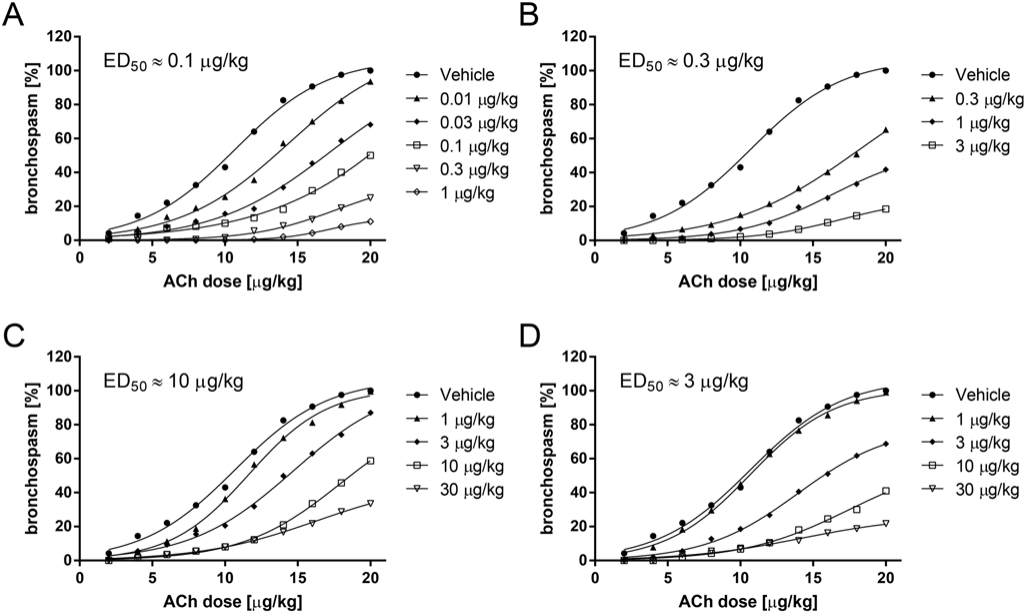
Therapeutic dose of β_2_-agonists in a model of acetylcholine-induced bronchoconstriction in anaesthetized guinea pigs.

In order to determine the time to onset of bronchoprotective activity and the therapeutic dose range of the different β_2_-AR agonists, the drugs were tested in a model of acetylcholine-induced bronchoconstriction in anaesthetized guinea pigs. For the experiments to determine time to onset of bronchoprotective activity the compounds were dissolved in a mixture of distilled water and ethanol (40:60, v/v) and administered utilizing the Respimat Soft Mist inhaler device connected to an endotracheal tube. Since all compounds reached their full efficacy within 15 minutes after application (Fig. 1) this time was chosen to determine the therapeutic dose. β_2_-AR agonists administered as a dry powder formulation with Respitose 2 hours before acetylcholine challenge inhibited acetylcholine-induced bronchoconstriction in a dose-dependent manner with ED_50_ values of 0.1 μg/kg for olodaterol, 0.3 μg/kg for formoterol, 10 μg/kg for salmeterol and 3 μg/kg for indacaterol (Fig. 2). Full bronchoprotection against acetylcholine (20 μg/kg) was observed with olodaterol at 1 μg/kg (89% bronchoprotection), 3 μg/kg formoterol (82% bronchoprotection) and 30 μg/kg indacaterol (78% bronchoprotection), while salmeterol at 30 μg/kg displayed less efficacy under these experimental conditions (67% bronchoprotection). However, the doses of formoterol, salmeterol and indacaterol could not be further increased to achieve full bronchoconstriction since higher doses induced a significant (>10% from baseline value) increase in heart rate (data not shown).

Time to onset of bronchoprotective activity of olodaterol (A), formoterol (B), salmeterol (C) and indacaterol (D) was tested in a model of acetylcholine-induced bronchoconstriction in anaesthetized Dunkin-Hartley guinea pigs. Compounds were administered intratracheally utilizing the Respimat Soft Mist inhaler device connected to an endotracheal tube. To address the onset of action, bronchoconstrictions were induced by acetylcholine (10 μg/kg i.v.) 1, 3, 5, 7, 10 and 20 min after drug inhalation. Data are given as mean of n = 4–7 animals per group.

Therapeutic dose range of bronchoprotective activity of olodaterol (A), formoterol (B), salmeterol (C) and indacaterol (D) was determined in a model of acetylcholine-induced bronchoconstriction in anaesthetized Dunkin-Hartley guinea pigs. Compounds were administered intratracheally as dry powder with Respitose. Increasing doses of acetylcholine from 2 to 20 μg/kg were intravenously applied 2 hours after drug administration. Data are given as mean of n = 4–7 animals per group.

### 2.2. Dry powder administration of olodaterol reduces citric acid-induced cough events in naïve guinea pigs

**Fig 3:**
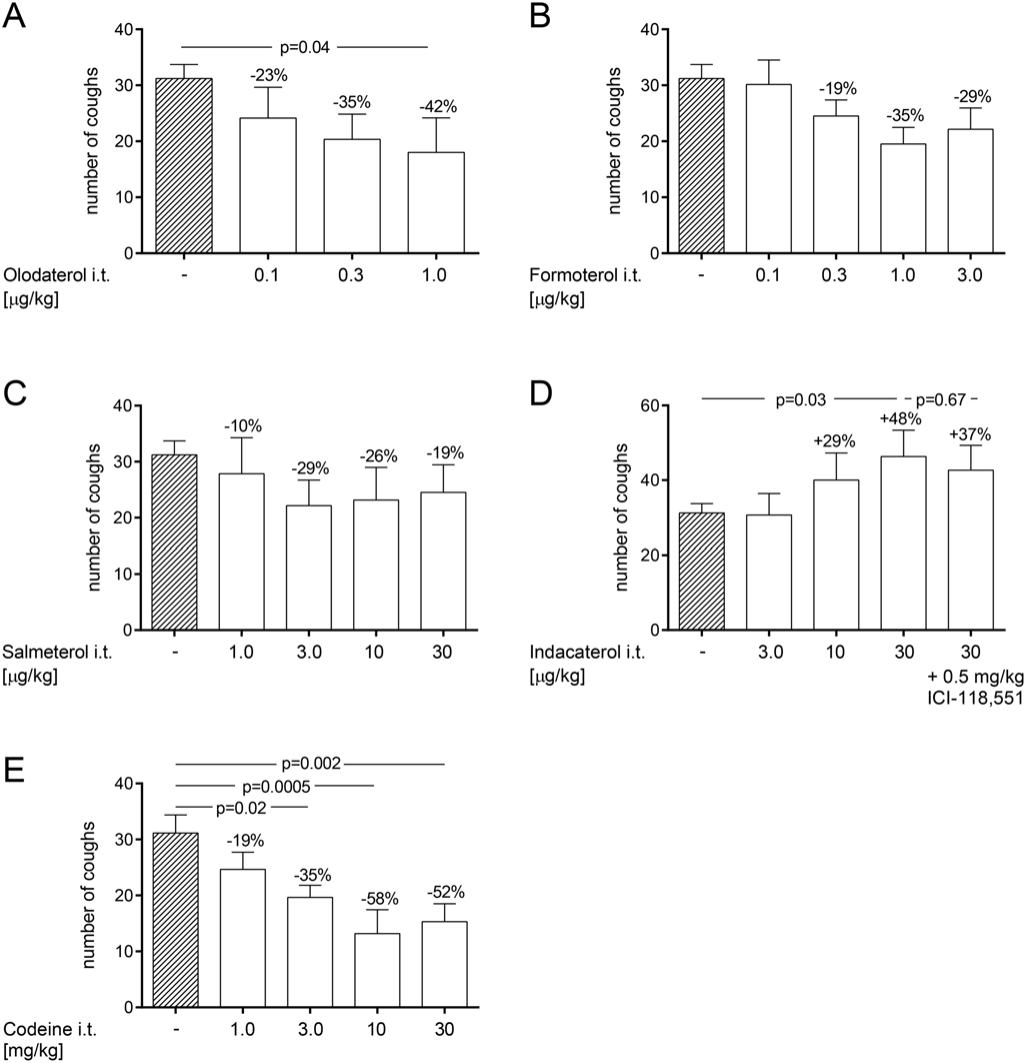
Effect of dry powder administration of β_2_-agonists on citric acid-induced cough in naïve guinea pigs.

In this experimental setup all test compounds were formulated as dry powders with Respitose and delivered using a powder inhaler device. Compounds were administered 15 minutes prior to the citric acid inhalation. Intratracheal administration of Respitose alone did not trigger any cough events (data not shown). However, administration of Respitose increased the number of citric acid-induced cough events from 12 to 31 coughs, 15 minutes after administration (data not shown). Olodaterol dose-dependently reduced the number of cough events with a maximal efficacy of 42% inhibition at 1 μg/kg (p < 0.05) (Fig. 3A). Formoterol and salmeterol also showed a trend towards reducing the number of cough events in this setting with a maximal efficacy of 35% inhibition at 1 μg/kg and 29% inhibition at 3 μg/kg, respectively (Fig. 3B and C). Indacaterol dose-dependently increased the number of cough events up to 48% at 30 μg/kg (p < 0.05) (Fig. 3D). Pre-treatment of guinea pigs with the β_2_-receptor antagonist, ICI-118,551, given at 0.5 mg/kg (i.p.) 30 minutes before intratracheal indacaterol administration did not block the pro-tussive effect of indacaterol (Fig. 3D). To validate the model codeine was formulated with Respitose and administered using the powder inhaler device. Codeine, which is an opiate used as a *cough* suppressant in the clinic, dose-dependently attenuated the number of citric acid-induced cough events with a maximal efficacy of 58% at 10 mg/kg (p < 0.001) (Fig. 3E).

Effect of dry powder administration of olodaterol (A), formoterol (B), salmeterol (C), indacaterol (D) and codeine (E) was determined in a model of citric acid-induced cough in naïve, conscious Dunkin-Hartley guinea pigs. All test compounds were formulated with Respitose and intratracheally delivered. Compounds were administered 15 min prior to the citric acid (0.4 M) inhalation. The β_2_-AR antagonist, ICI-118,551, was administered intraperitoneally 30 minutes before indacaterol application. The number of cough events was counted for 15 min during the citric acid inhalation. p-values represent significant difference compared with the vehicle-treated control group receiving Respitose using one-way ANOVA followed by Fisher’s LSD post-hoc test. For the comparisons a method for ordered alternatives was used to adjust for multiple testing. Data are given as mean ± SEM of n = 6 animals per group except for the negative control group which consists of n = 36 animals.

### 2.3. Dry powder administration of olodaterol dose-dependently reduces citric acid-induced cough events in OVA-sensitized guinea pigs

**Fig 4:**
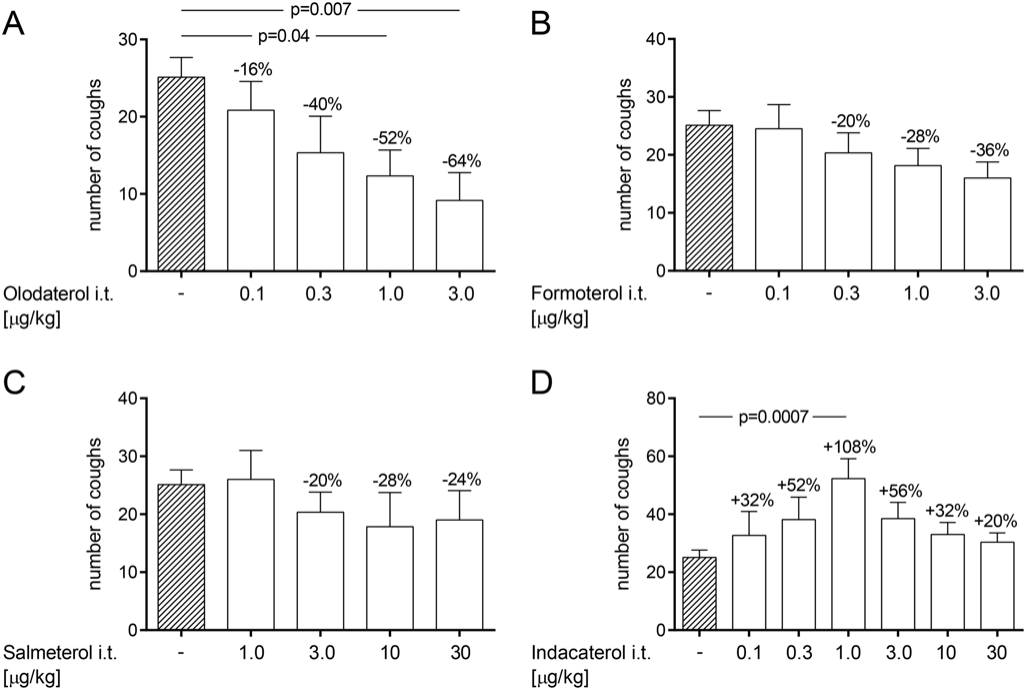
Effect of dry powder administration of β_2_-agonists on citric acid-induced cough in OVA-sensitized guinea pigs.

To simulate the increased cough reflex sensitivity found in asthmatic patients guinea pigs were sensitized to OVA. Successful sensitization was demonstrated by measuring serum-specific IgE against OVA by ELISA in OVA-immunized guinea pigs versus naïve guinea pigs. OVA-specific serum IgE levels increased from 10.3 ng/ml in naïve animals to 287.9 ng/ml in OVA-sensitized animals (data not shown). The number of citric acid-induced coughs increased from 12 in naïve animals to 24 in OVA-sensitized and challenged animals (data not shown). After Respitose treatment OVA-sensitized and challenged animals coughed 25 times. This response was dose-dependently inhibited by olodaterol, with a maximal inhibition of 6400A0% at 3 μg/kg (p<0.01) (Fig. 4A). Formoterol and salmeterol also reduced the number of cough events in this setting with a maximal efficacy of 36% inhibition at 3μg/kg and 28% inhibition at 10μg/kg, respectively (Fig. 4B and C). In contrast, indacaterol increased the number of cough events up to 108% at 1 μg/kg (p<0.001). The number of cough events induced by indacaterol exhibited a bell-shaped curve to increasing doses of indacaterol with a maximum at 1 μg/kg (Fig. 4D).

Effect of dry powder administration of olodaterol (A), formoterol (B), salmeterol (C) and indacaterol (D) was determined in a model of citric acid-induced cough in OVA-sensitized, conscious Dunkin-Hartley guinea pigs. Animals were immunized on day 1 and 2 by subcutaneous injection of OVA. On day 15 animals were exposed to an OVA aerosol. On day 16, all test compounds were formulated with Respitose and intratracheally delivered 15 min prior to the citric acid (0.4 M) inhalation. The number of cough events was counted for 15 min during the citric acid inhalation. p-values represent significant difference compared with the vehicle-treated control group receiving Respitose using one-way ANOVA followed by Dunnett’s post-test. Data are given as mean ± SEM of n = 6 animals per group except for the negative control group which consists of n = 18 animals.

## Discussion

In our study we compared the efficacy of different clinically relevant β_2_-AR agonists, given intratracheally formulated as a dry powder, with respect to their antitussive properties in a model of citric acid-induced cough in two different settings, namely in naïve and in OVA-sensitized conscious guinea pigs. The novel once-daily β_2_-AR agonist olodaterol demonstrated dose-dependent and statistically significant antitussive properties both, in naïve and OVA-sensitized animals. Furthermore, olodaterol seemed to inhibit citric acid-induced cough events more potently and with a greater maximal efficacy in OVA-sensitized guinea pigs compared to naïve animals. The two twice-daily β_2_-AR agonists, formoterol and salmeterol, which have been used as bronchodilators in the clinical practice for years, both showed a trend towards reducing the number of citric acid-induced cough events in both settings. On the contrary, the once-daily β_2_-AR agonist indacaterol increased the number of citric acid-induced cough events both in naïve and even more potently in OVA-sensitized guinea pigs. The described effects on cough were observed at comparable bronchoprotective doses for all β_2_-AR agonist as determined in a model of acetylcholine-induced bronchoconstriction.

Our findings, which show that olodaterol, formoterol and salmeterol exhibit antitussive properties, are in agreement with the literature as numerous pre-clinical studies demonstrated antitussive properties of β_2_-AR agonists in different experimental settings. For example, the short-acting β_2_-AR agonist salbutamol given subcutaneously inhibited both allergic and capsaicin-induced cough [26]. Procaterol given intraperitoneally reduced the cough response to capsaicin in conscious guinea pigs [27]. Lewis et al. showed that the β_2_-AR agonist terbutaline, given either subcutaneously or orally, reduced the number of exacerbated cough events to citric acid or capsaicin following cigarette smoke exposure in conscious guinea pigs [28]. Furthermore, Freund-Michael et al. demonstrated that terbutaline given intraperitoneally reduced the number of capsaicin- and citric acid-induced cough events in naïve guinea pigs [10]. However, none of the studies described above tested the effect of the β_2_-AR agonists on cough after intratracheal application of the test substance which is the most relevant route of application taken into account that preferred *treatment* of respiratory diseases is with *inhalation therapy*. Thus, our data do not only confirm previous observations in guinea pigs but extend our knowledge to the antitussive properties of β_2_-AR agonists after intratracheal application.

Whether the reduction in the number of coughs can be attributed directly to β_2_-ARs expressed on peripheral terminals of airway sensory nerves or whether the effect is due to bronchodilation mediated via β_2_-ARs expressed on airway smooth muscle cannot be stated on the basis of our data. However, Freund-Michel et al. demonstrated in a recent publication that the β_2_-AR agonist effect on cough is probably mediated by β_2_-ARs expressed on vagal nerve endings activation of which induces hyperpolarization of the neuron membrane thereby inhibiting sensory nerve activation and the cough reflex [10].

Regarding the pro-tussive effect of indacaterol limited pre-clinical data is available. In the Australian Public Assessment Report for indacaterol data are cited describing that indacaterol elicited a weak but statistically significant cough response in conscious guinea pigs when administered via the inhalational route as a nebulized 2.0 mg/ml solution in 100% ethanol [29]. These data are in agreement with our data that showed an increased cough response to citric acid after intratracheal application of indacaterol. Additionally, we were able to demonstrate that the indacaterol-induced increase in cough is not mediated via the β_2_-AR, since pre-treatment with the selective β_2_-receptor antagonist ICI-118,551 did not block the pro-tussive effect of indacaterol. Yet, the molecular mechanism underlying the pro-tussive effect of indacaterol is not clear. One explanation could be that indacaterol, by activating the Transient Receptor Potential cation channel, subfamily A, member 1 (TRPA1), which is expressed by vagal neurons, might activate lung vagal C-fibres, thereby inducing cough. This speculation is based on the fact that in CHO cells, transfected with the human TRPA1, indacaterol showed weak excitatory activity and that activation of vagal C-fibres from guinea pig lungs was observed at 100 μM indacaterol maleate [29]. This could also explain the bell-shape curve that was observed with increasing indacaterol doses in OVA-sensitized guinea pigs since it is a known phenomenon for TRPA1 agonists that they quickly induce desensitization [30]. However, whether the weak agonistic effect of indacaterol at the TRPA1 channel accounts for its pro-tussive effect is questionable since salmeterol is a more potent stimulant of this ion channel than indacaterol but pro-tussive activity has not been described for salmeterol [31]. Another hypothesis might be that low doses of indacaterol could activate C-fibres from the jugular ganglia involved in coughing while increasing doses of indacaterol may activate C-fibres from the nodose ganglia which are supposed to reduce cough [32]. Furthermore, the pro-tussive effect of indacaterol might result from a more effective β_3-_receptor activation which was demonstrated to enhance the excitability of rat vagal chemosensitive neurons [33]. Particularly, since indacaterol has the smallest β_3_/β_2_-ratio of all four tested compounds [24,34]. Nevertheless, the four β_2_-agonists tested in our study have a rather low binding affinity to the β_3_-receptor (pKi values between 5.3 and 5.6) [24,34], calling this hypothesis into question.

Differences seen in OVA-immunized compared to naïve guinea pigs might stem from a different receptor expression profile in naïve and sensitized animals. One might speculate that OVA-sensitization might increase β_2_-AR expression, thus, explaining the greater potency and maximal efficacy of olodaterol in OVA-sensitized guinea pigs compared to naïve animals. This hypothesis would be in agreement with studies showing that allergen challenge induces the expression of another receptor associated with cough, namely the Transient Receptor Potential Vanilloid subtype 1 (TRPV1) in the airways [35,36]. However, whether OVA challenge induces the expression of β_2_-ARs on airway sensory nerves has to our knowledge not been investigated.

Comparing our data to the clinical situation is challenging since despite considerable pre-clinical evidence, controversial clinical data on the antitussive properties of β_2_-AR do exist. One reason for this discrepancy might originate from the fact that pre-clinical studies often use different application routes, dosing regimens and formulations compared to the clinical situation. While some clinical studies show a reduction in cough events after inhalation of β_2_-AR agonists [11–17] others failed to demonstrate this effect [18–22]. An explanation why a dominant antitussive property of β_2_-AR agonists has not been uncovered in clinic trials until now might be that many studies were conducted in healthy volunteers rather than in patients with pathological cough [37]. Furthermore, in many studies with a negative outcome cough was not the primary endpoint and β_2_-AR agonist doses were not geared to show antitussive effects [10]. Furthermore, no objective measurement of cough was possible since objective cough monitoring devices have only recently been become available.

For the once-daily inhaled, long-acting β_2_-adrenergic receptor agonist indacaterol mild transient cough has been shown to be a relatively common adverse effect in the clinic [38,39]. In a 28-day randomized, placebo-controlled clinical trial in COPD patients, indacaterol increased the incidence of cough by 14.7% and 28.4% in the indacaterol 400 and 800 μg groups, respectively, compared with no patients in the placebo group [40]. In a 12-week Phase III study in patients with moderate-to-severe COPD cough was recorded with an average incidence of 17.8% with indacaterol (150 μg once-daily) compared to 3.3% with placebo [41].

In summary our data further substantiate previous pre-clinical experiments in guinea pigs by showing that olodaterol, formoterol and salmeterol reduce cough after intratracheal dry powder application. Furthermore, our pre-clinical model of citric acid-induced cough in guinea pigs mirrors clinical data which demonstrated antitussive properties of the long-acting β_2_-AR agonists formoterol and salmeterol and pro-tussive effects of indacaterol [11,17,40,41]. Additionally, we demonstrated for the first time the antitussive efficacy of the once-daily β_2_-AR agonist olodaterol.
